# Venous thromboembolic events during warm autoimmune hemolytic anemia

**DOI:** 10.1371/journal.pone.0207218

**Published:** 2018-11-08

**Authors:** Sylvain Audia, Benoit Bach, Maxime Samson, Daniela Lakomy, Jean-Baptiste Bour, Bénédicte Burlet, Julien Guy, Laurence Duvillard, Marine Branger, Vanessa Leguy-Seguin, Sabine Berthier, Marc Michel, Bernard Bonnotte

**Affiliations:** 1 Department of Internal Medicine and Clinical Immunology, Constitutive Referral Center for Autoimmune Cytopenias, University Hospital, Dijon, France; 2 Immunology laboratory, University Hospital, Dijon, France; 3 Department of Virology, University Hospital, Dijon, France; 4 Hematobiology, University Hospital, Dijon, France; 5 Biochemestry laboratory, University Hospital, Dijon, France; 6 French National Blood Service, Dijon, France; 7 Department of Internal Medicine, Referral Center for Autoimmune Cytopenias, Henri Mondor University Hospital, Creteil, France; Institut d'Investigacions Biomediques de Barcelona, SPAIN

## Abstract

Thrombotic manifestations are a hallmark of many auto-immune diseases (AID), specially of warm autoimmune hemolytic anemia (wAIHA), as 15 to 33% of adults with wAIHA experience venous thromboembolic events (VTE). However, beyond the presence of positive antiphospholipid antibodies and splenectomy, risk factors for developing a VTE during wAIHA have not been clearly identified. The aim of this retrospective study was to characterize VTEs during wAIHA and to identify risk factors for VTE. Forty-eight patients with wAIHA were included, among whom 26 (54%) had secondary wAIHA. Eleven (23%) patients presented at least one VTE, that occurred during an active phase of the disease for 10/11 patients (90%). The frequency of VTE was not different between primary and secondary AIHA (23.7 *vs*. 19.2%; *p* = 0.5). The Padua prediction score based on traditional risk factors was not different between patients with and without VTE. On multivariate analysis, total bilirubin ≥ 40 μmol/L [odds ratio (OR) = 7.4; *p =* 0.02] and leucocyte count above 7x10^9^/L (OR = 15.7; *p =* 0.02) were significantly associated with a higher risk of thrombosis. Antiphospholipid antibodies were screened in 9 out the 11 patients who presented a VTE and were negative. Thus, the frequency of VTE is high (23%) during wAIHA and VTE preferentially occur within the first weeks of diagnosis. As no clinically relevant predictive factors of VTE could be identified, the systematic use of a prophylactic anticoagulation should be recommended in case of active hemolysis and its maintenance after hospital discharge should be considered. The benefit of a systematic screening for VTE and its procedure remain to be determined.

## Introduction

Few studies have reported an increased risk of venous thromboembolism (VTE) in autoimmune diseases (AID) [1–4]. Various risk factors are suspected to be involved such as the presence of antiphospholipid antibodies [5,6], the use of steroid pulses [7] and chronic inflammation leading to some endothelial dysfunctions and increased expression of tissue factor [5]. The increased risk of VTE during wAIHA was first noticed in the 60’s by Allgood, who reported the occurrence of pulmonary embolism in 5 out of 47 AIHA, among which 4 were fatal [8]. A higher risk of thrombosis in patients with auto-immune hemolytic anemia (AIHA) as compared to other AIDs has been reported, particularly within the 90 days following disease onset [4]. Although the causes of thrombosis are multifactorial in these patients, the release of some red blood cell components may contribute to this prothrombotic state, notably the increase in free hemoglobin level which could diminish the serum concentration of nitric oxide (NO) which inhibits platelet aggregation [9–11]. In the literature, the relative risk of VTE in patient with AIHA is around 2.6 [12]. Risk factors, such as splenectomy [8,13] or the presence of antiphospholipid antibodies [6] have been suggested. In the largest series of primary AIHA including 308 patients, a thrombotic event, either venous or arterial, was detected in 11% and was associated with a severe onset of the disease, as represented by a hemoglobin level below 80 g/L at diagnosis and a higher LDH level. Splenectomy was associated with an increased risk of thrombosis while the presence of anticardiolipin antibodies or lupus anticoagulant were not [13].

A recent study reported the clinical and biological characteristics of patients with wAIHA and thrombosis [14]. Neither the traditional risk factors for thromboembolism evaluated by the Padua prediction score, nor the clinical and biological parameters at diagnosis could predict the occurrence of VTE, except for the nadir of hemoglobin level during the follow-up, which was lower in patients with VTE.

The aim of our study was to better describe the characteristics of VTE occurring in patients with wAIHA and to identify risks factors that could help to define preventive measures.

## Materials and methods

### Patients

Medical records of all patients seen at our referral University Hospital Center between March 2006 and March 2016 for hemolytic anemia according to the diagnosis-related group (DRG) medical information system (PMSI) were retrospectively reviewed. The study was approved by the institutional review board of the University Hospital of Dijon and the local ethics committee (Comité de Protection des Personnes Est I), who waived the requirement for informed consent.

The inclusion criteria were: 1) age >18 years, 2) a diagnosis of wAIHA based on a hemoglobin level <120 g/L, with a haptoglobin level <0.2 g/L and a positive direct antiglobulin test (DAT) with an IgG ± C3d pattern. Exclusion criteria were: the presence of numerous schistocytes, a diagnosis of hereditary hemolytic anemia, a negative DAT or positive DAT with a C3d pattern alone and/ or the presence of a high titer of cold agglutinins.

wAIHA were defined as primary or secondary: the diagnosis of secondary wAIHA was retained when an underlying myeloid or lymphoid malignancy, another AID such as systemic lupus (SLE) or a concomitant and potentially causal infection was present. The diagnoses of underlying disorders were based on consensual international criteria for SLE [15], dermatomyositis [16], lymphomas [17], myelodysplastic syndromes [18], multiple myeloma [19] and myeloproliferative neoplasms [18].

Administrative data (age, gender), clinical presentation at diagnosis (asthenia, dyspnea, jaundice, angina), biological data (hemoglobin level (g/L), mean corpuscular volume (MCV, μ^3^), LDH level (UI/L), bilirubin level (μmol/L), DAT pattern, leukocyte count (x10^9^/L), lymphocyte count (x10^9^/L), antinuclear antibodies (ANA), immunoelectrophoresis, levels of C3 (g/L), C4 (g/L), total complement activity (CH50, U/mL), results of bone marrow biopsy whenever performed and results of radiological investigations (adenopathy (>10 mm), splenomegaly, or hepatomegaly on CT-scan) were collected.

The different treatments for wAIHA and the rates and patterns were also analyzed. A complete response (CR) was defined as a hemoglobin level >120 g/L without any transfusion and no ongoing hemolysis. Partial response (PR) was defined by an increase in hemoglobin level of at least 20 g/L from the baseline level with persistent of hemolysis.

### VTE evaluation

The Padua prediction score, that is validated to determine the risk of venous thromboembolism occurrence for patients hospitalized in medical units, was determined [20,21]. Parameters included in this score are: active cancer or chemotherapy or radiotherapy in the past 6 months, previous VTE with the exclusion of superficial thrombosis, bedrest >3 days, known thrombophilia, surgery or trauma in the previous month, age >70, cardiac or respiratory failure, acute myocardial infarction or stroke, obesity (BMI >30 kg/m^2^), and ongoing hormonal treatment. The use of an antiplatelet therapy or anticoagulant was also recorded.

VTE, *i*.*e*. deep venous thrombosis (DVT) or pulmonary embolism (PE), confirmed by venous ultrasound or CT-angiography respectively, were recorded. Thrombosis of the portal system following splenectomy (n = 2) were not considered in the analysis of the results. Wells score calculated for patients with DVT or PE was recorded. Screening for thrombophilia were also recorded (lupus anticoagulant or antiphospholipid antibodies, protein S, protein C and/or antithrombin III levels, mutations of factor II or factor V, and JAK2 V617F mutation).

Frozen sera were available at the time of wAIHA diagnosis for 7 patients with VTE and 22 without to retrospectively measure the level of free hemoglobin by spectrophotometry using the benzidine method [22], while the level of soluble CD163 (ELISA Kit, Invitrogen) and nitrites/nitrates (nitrite/nitrate assay kit colorimetric, Sigma-Aldrich) were measured following manufacturers’ instructions.

### Statistical analysis

Due to the size of the cohort, the absence of Gaussian distribution or the absence of homoscedasticity, non-parametric tests were used to compare data from wAIHA patients with VTE to those of patients without VTE. Quantitative data are reported as medians [interquartile rate (IQR)] and were compared using the Mann-Whitney test. Qualitative variables are reported as frequencies and were compared using the Chi^2^ test or Fisher’s exact test when groups contained less than five patients. *P*<0.05 was considered significant. Analyses were performed with SPSS (IBM). A multivariate analysis was performed with step-to-step regression, including variables with *p*-value below 0.2 on univariate analysis.

## Results

### Population

**Fig 1:**
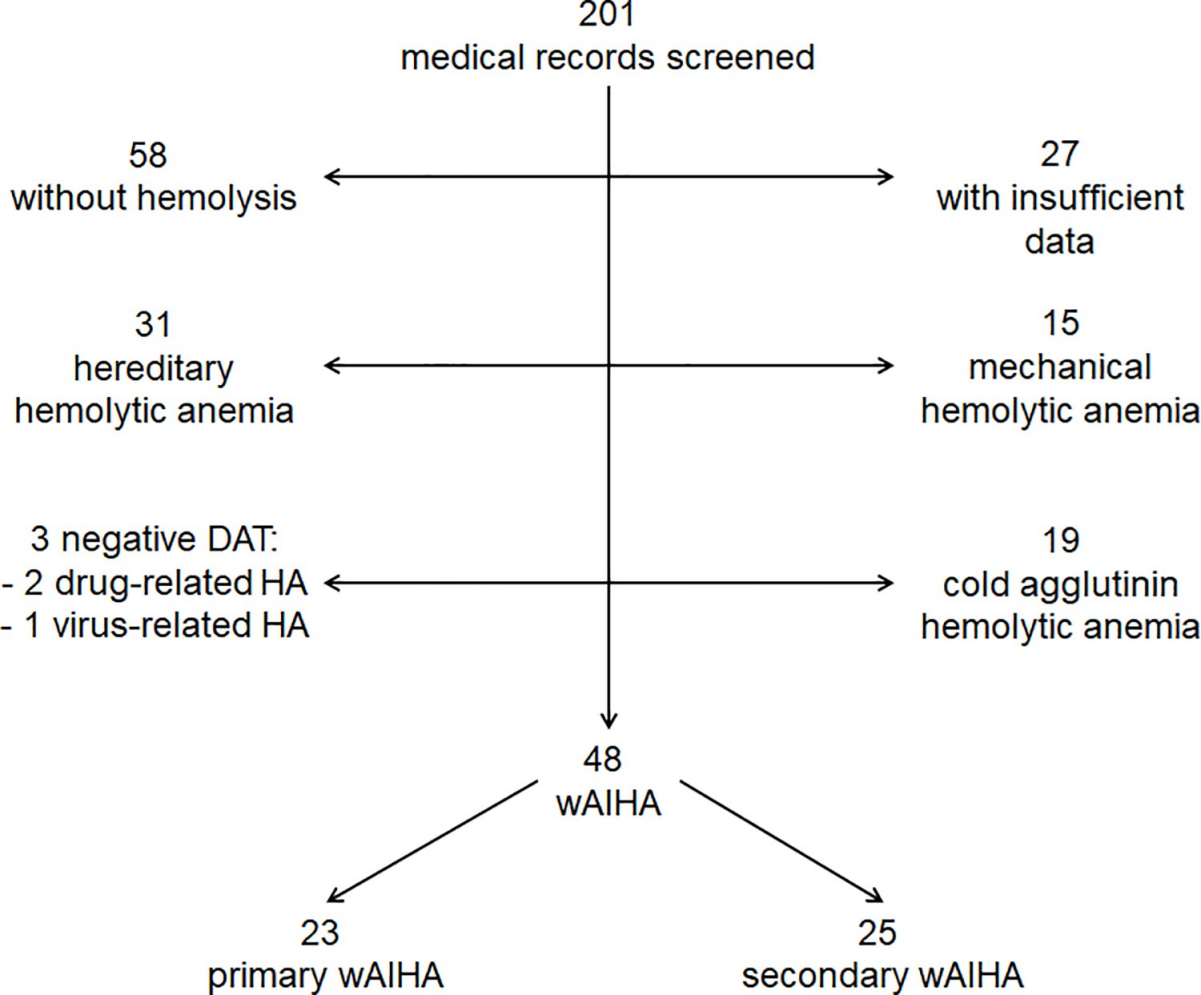
Flowchart of patients.

In total, 201 patients with hemolytic anemia were identified among whom 153 were excluded after analysis of medical records (Fig 1). The main causes of exclusion were: hereditary hemolytic anemia (n = 31), mechanical hemolytic anemia (n = 15), cold agglutinins (n = 19) or negative DAT (n = 3). Twenty-seven patients were also excluded because of missing data, whereas 58 had no features of hemolysis.

Forty-eight patients (50% female, median age of 65 years) with wAIHA were included, 23 of whom (48%) had primary wAIHA (S1 Table). The underlying diseases or conditions found in secondary wAIHA (n = 25) were the following: lymphoma (n = 14, including 6 patients with chronic lymphocytic leukemia), other autoimmune diseases (n = 5: systemic lupus (n = 3), dermatomyositis (n = 1), cryoglobulinemia (n = 1)), infections (n = 2: CMV (n = 1) and EBV (n = 1)), common variable immunodeficiency (n = 1), myeloproliferative neoplasm (n = 1) and myelodysplastic syndrome (n = 2).

All patients received at least one treatment-line for wAIHA: steroids were used as first line therapy in almost all patients (98%) with a response rate of 85% (CR: 47%). Chemotherapy-based treatments combined with steroids were used as first line therapy for 27% of secondary wAIHAs, due to lymphoma-associated wAIHA. A second line therapy was required in 75%, most particularly for secondary wAIHA (88%). Rituximab was used in 42% of cases with an overall response-rate (CR + PR) of 79%, whereas splenectomy was performed in 25% of the patients, with a response achieved in only 3 patients (33%). Finally, a third line of treatment was required in 38% of patients, particularly for secondary wAIHA (43%).

### Frequency of VTE and characteristics of patients with VTE

After a median follow-up of 24.8 months (10.1–62.9) from wAIHA diagnosis, 11 out of 48 patients (23%) had presented at least one VTE. The characteristics of the patients who had a VTE are summarized in Table 1. Six patients presented with either PE (n = 1) or DVT (n = 5) while 5 patients were diagnosed with both concomitantly.

**Table 1:** Characteristics of patients with VTE.

# patient	Gender	Age at wAIHA diagnosis	Hemolysis at VTE	Type of wAIHA	Hb level at AIHA diagnosis (g/dL)	Hb level at VTE diagnosis (g/dL)	Time between diagnosis of wAHAI and VTE (weeks)	VTE	Risk factors of VTE	Padua score at wAIHA diagnosis	Padua score at VTE diagnosis	APL	In/out patient	Days of hospitalization / days after discharge from previous hospitalization at VTE occurrence	Cause of hospitalization	Symptoms of VTE	Thrombotic prophylaxis	Anticoagulant and duration	Relapse of VTE
1	F	81	+	Primary	8,2	8,2	0	PE	Obesity	3	3	ND	Out	D1	Dyspnea = > PE diagnosis	Dyspnea	None	VKA, long-term	0
2	F	69	+	Primary	7,4	7,4	0,5	PE+DVT	0	1	1	0	Out	D1	Anemia	Calf pain	None	VKA, 1 year	0
3	M	63	+	Primary	4,9	5,7	1	DVT	Obesity	1	1	0	In	D2	Anemia	None (systematic screening)	None	Rivaroxaban, 3 months	0
4	F	54	+	Primary	5	4,8	3,5	PE+DVT	Bed rest (due to anemia)	5	8	0	Out	D1	Acute respiratory failure = > PE diagnosis	Dyspnea	None	VKA, 6 months	0
5	F	42	+	Primary	8,9	6	4	DVT	0	0	0	ND	In	D7	Anemia	Lower limb edema	Prophylactic LMWH	VKA, long-term	2
6	M	76	+	Secondary (low grade NHL)	7,0	7	6	DVT	0	2	5	0	Out	D1 / D28 discharge	Anemia	Lower limb edema	VKA (INR 1.5)	VKA, long-term	0
7	M	22	-	Secondary (IIM)	12,3	14	7	PE+DVT	Bed rest + IVIg (2g/kg for IIM)	3	3	0	Out	D1 / D7 discharge	Chest pain = > PE diagnosis	Chest and calf pain	None	VKA, 6 months	0
8	F	83	+	Primary	7,3	8,5	7	PE+DVT	0	0	0	0	In	D7	Anemia	Persisting dyspnea	None (referral from another hospital)	VKA, 6 months	0
9	M	32	+	Primary	5,4	9,2	7	PE+DVT	IVIg (2g/kg for ITP)	0	0	0	Out	D1 / D15 discharge	Calf pain, dyspnea	Calf pain, dyspnea	None	VKA, 1 year	0
10	M	86	+	Secondary (low grade NHL)	7,4	9,8	18	DVT	0	2	5	0	In	D15	Pancytopenia	Lower limb edema	None (thrombocytopenia <30 G/L)	VKA, 6 months	0
11	M	54	+	Secondary (HCV, cryoglobulinemia)	5,6	6,6	23	DVT	Nephrotic syndrome	3	3	0	Out	D1 / D7 discharge	Anemia	Calf pain	None	VKA, 6 months	1

**APL**: antiphospholipid antibodies, **DVT**: deep vein thrombosis, **F**: female, **Hb**: hemoglobin, **HCV**: Hepatitis C Virus, **IIM**: Idiopathic Inflammatory Myopathy, **IVIg**: intravenous immunoglobulins, **LMWH**: Low Molecular Weight Heparin, **M**: male, **ND**: not determined, **NHL**: Non-Hodgkin Lymphoma, **PE**: pulmonary embolism, **VKA**: vitamin K antagonist, **VTE**: venous thromboembolism, **wAIHA**: warm autoimmune hemolytic anemia

At the time of VTE, active hemolysis was present in 10/11 patients (91%). In 8 out of these 10 patients, the VTE occurred within 2 months after the diagnosis of wAIHA, within the the first month for 4 patients. Seven of these patients had primary wAIHA and 4 had secondary wAIHA (low grade B-cell non-Hodgkin lymphoma (n = 2), idiopathic inflammatory myopathy (dermatomyositis) and cryoglobulinemia with hepatitis C infection). The diagnosis of VTE was performed within the 2 days of hospitalization in 8 cases. Of note, 4 of them have been discharged from the hospital few weeks before (1 week for 2, 2 and 4 weeks for the others), without prophylactic anticoagulation, except for one patient who was on vitamin K antagonist for atrial fibrillation but with an INR below therapeutic range. For 3 patients, VTE occurred while hospitalized for anemia. One patient (#5) was on prophylactic low molecular weight heparin, while the 2 others had no thromboprophylaxis: one patient was referred from another hospital where she did not receive thromboprophylaxis for unknown reason (#8), while the other was not treated because of thrombocytopenia, despite a Padua score of 5 (#10). It is worth noticing that 2 out of 8 outpatients who did not receive thromboprophylaxis despite a high Padua score, *i*.*e*. >4 (#4 and #6). Antiphospholipid antibodies, screened in 9 out of 11 patients, were not detected. Lupus anticoagulant (LA) screened with aPTT was normal in all patients. VTE was unprovoked for six patients, while 2 patients were treated with intravenous immunoglobulin infusion prior to VTE (one for ITP (Evans syndrome), one for idiopathic inflammatory myopathy), one had a nephrotic syndrome (hepatitis C membranous glomerulonephritis, with albuminemia at 27 g/L and proteinuria at 3,27 g/d at VTE), and 2 have been bedridden for more than 3 days before hospitalization (one because of idiopathic inflammatory myopathy, the other because of the worsening of anemia).

Notably, 4 patients were hospitalized because a VTE was suspected. For the others, hospitalization was due to anemia, but symptoms of VTE were detected at admission for 3 out of 7 patients or appeared during hospitalization for 3 others. For one patient, the diagnosis of DVT was performed after a systematic screening.

The median DVT Wells score was 4 (IQR:1–5) and the median PE Wells score was 4 (2–7). Of note, none of the patients with VTE had a measurement of D-dimers.

Two patients had several VTE. Each episode occurred during a flare or a relapse of wAIHA when the patients were no longer on curative anticoagulation therapy. A triggering factor was identified in both cases: thrombosis of the upper limb following the peripheral insertion of a central catheter in one patient, and sepsis in the other.

### Comparison of wAHAI patients with or without VTE

There was no difference regarding age, gender or type of wAIHA (primary or secondary) between patients with or without VTE (Table 2). Of note, the frequency of VTE was similar between primary and secondary wAIHA (27.3% *vs*. 19.2% respectively; *p* = 0.5), as was the type of VTE [DVT (21.7% *vs*. 15.4%; *p* = 0.9) and PE (17.4% *vs*. 7.7%; *p* = 0.4)].

**Table 2:** wAIHA baseline characteristics in the whole cohort and comparison between patients with or without VTE.

		Overall AIHA n = 48	AIHA without VTE n = 37	AIHA with VTE n = 11	*p-*value
**Epidemiology at wAIHA diagnosis**	Number of females; n (%)	24 (50)	19 (51. 4)	5 (45.5)	0.73
	Median age	65 [44–78]	64.79 [32.9]	63.43 [39]	0.79
	Primary AIHA; n (%)	23 (48)	16 (43.2)	7 (63.6)	0.31
	Median Padua score	1 [0–3.75]	1 [0–3.5]	2 [0–3]	0.97
	Median Padua score (at VTE diagnosis)	-	-	3 [0–5]	0.56
	Number of deaths; n (%)	8 (16.6)	6 (16.2)	2 (18.2)	1
	Antiplatelet therapy; n (%)	7 (14.3)	5 (13.5)	1 (9.1)	0.49
	VKA; n (%)	14 (28.6)	7 (18.9)	1 (9.1)	0.40
**Clinical features at wAIHA diagnosis**	Dyspnea; n (%)	17 (36.1)	12 (32.1)	6 (54)	0.30
	Icterus; n (%)	8 (16.2)	2 (7.1)	5 (44.4)	0.02
**Biological parameters at wAIHA diagnosis**	Hemoglobin (g/L)	73 [63–90]	72 [63–88.5]	74 [56–89]	0.90
	Reticulocytes (x10^9^/L)	182 [121–287]	157.5 [114–249]	287.9 [147–341]	0.06
	Platelets (x10^9^/L)	237 [155–339]	228 [155–310]	283 [165–364]	0.56
	Leukocytes (x10^9^/L)	7.9 [5.6–12.35]	7.3 [5.4–10.5]	11.9 [8.6–18.1]	0.02
	LDH level >normal range; n (%)	37 (77)	28 (75.7)	9 (81.8)	1
	Total bilirubin (μmol/L)	33.5 [22.5–45]	31 [25–39]	41 [32–47.5]	0.04
	Total bilirubin≥40; n (%)	15 (31)	8 (21.6)	7 (63.6)	0.02
	Free bilirubin (μmol/L)	32 [26.3–41]	31 [24.5–39.5]	39.5 [31.5–47.2]	0.09
	Free bilirubin ≥35; n (%)	15 (31)	9 (24.3)	6 (54.5)	0.07
	CRP (mg/L)	10.3 (3.7–25.3)	9.8 (4.5–25.4)	10.9 (3–25.9)	0.9
	CRP (mg/L) at VTE diagnosis	-	-	13.5 (6.9–39.8)	0.5
	CH50 <normal range *22/48*; n (%)	4 (9.1)	2 (5.4)	0 (0)	0.52
	Low C3 <normal range *26/48*; n (%)	12 (24)	2 (5.4)	3 (27.3)	0.10
	Low C4 <normal range *26/48*; n (%)	12 (24)	5 (13.5)	1 (9.1)	0.68
	Abnormality of lymphoid lineage on bone marrow aspirate *27/48*; n (%)	11 (23.1)	8 (22.7)	3 (25)	0.68
**Imaging**	Splenomegaly *36/48*; n (%)	12 (26.7)	9 (25)	3 (37.5)	0.39
	Hepatomegaly *36/48*; n (%)	7 (15.6)	4 (10.7)	3 (37.5)	0.11
	Adenopathy *36/48*; n (%)	7 (15.6)	4 (10.7)	3 (37.5)	0.11

**CRP**: C reactive protein, **LDH**: lactate dehydrogenase, **VKA**: vitamin K antagonists. Quantitative variables are reported as medians [interquartile rate]. Qualitative variables are reported as numbers (frequencies); n (%).

The Padua prediction scores were similar between patients without and those with VTE, either at wAIHA diagnosis and at VTE diagnosis [1 (0–3.5) *vs*. 2 (0–3); *p* = 0.97 and 1 (0–3.5) *vs*. 3 (0–5); *p* = 0.56 respectively]. Neither the proportion of patients on long-term antiplatelet therapy nor the percentage of patients treated with anticoagulant at the diagnosis of wAIHA was different in the two groups. Of note, although the frequencies of stroke and myocardial infarction prior to the diagnosis of wAIHA, collected as part of the Padua score, were not different between patients with or without VTE (1/11 *vs*. 6/37; *p* = 1), a stroke occurred during follow-up in 2 patients of the VTE group, while none was recorded in the non VTE group.

All patients reported fatigue at wAIHA diagnosis, while the presence of dyspnea was not different between groups (32.1% *vs*. 54%; *p* = 0.3). Interestingly, jaundice at diagnosis was recorded more frequently in the VTE group (44.4% *vs*. 7.1%; *p* = 0.02).

A total of 8 (16.6%) patients died during the follow-up: infection was the most frequent cause of death (5 patients). Importantly, the occurrence of a VTE does not affect the mortality rate.

The level of hemoglobin at diagnosis was not different between both groups (72 (63–88.5) *vs*. 74 (56–89) g/L; *p* = 0.9). Although non-significant, the reticulocyte count at wAIHA onset tended to be higher in the VTE group (287.9 (147–341) *vs*. 157.5 (114.75–249.25) x10^9^/L; *p* = 0.06). The level of total bilirubin was higher in the VTE group [31 (25–39] *vs*. [41 (32–47.5) μmol/L; *p* = 0.04]. The median leukocyte count was greater in case of VTE [11.9 (8.6–18.1) *vs*. 7.3 (5.4–10.5) x10^9^/L; *p* = 0.02], which did not rely on the primary or secondary nature of wAIHA (not shown). A leukocytosis >10 G/L was observed in 19 patients and attributed to infection (n = 4), lymphoproliferative neoplasm (n = 4) or to bone marrow hyperstimulation secondary to AIHA (n = 11) as no other causes were identified. C-reactive protein (CRP) concentration was not different between patients without or with VTE, either at wAIHA diagnosis or at VTE diagnosis. Moreover, the leukocyte count was not correlated with CRP level (r = 0.7, derived by Spearman correlation test). No difference was observed concerning LDH, complement levels and bone marrow findings.

Thirty-six patients had a CT-scan of the chest and abdomen and pelvis: the presence of splenomegaly, adenopathy or hepatomegaly were not different between groups.

Steroids were used as first line therapy in 45 out of 48 patients, and were initiated at 1 mg/kg/d in 23 cases, at 1.5 mg/kg/d in 5 cases and at 2 mg/kg/d in 17 cases, which was not different between patients with or without VTE (not shown). The use of different starting doses was due to the evolution of guidelines over the time. Of the 11 patients treated with rituximab, only 2 had a VTE that was not attributed to rituximab as it occurred 4 and 6 months after infusion.

A multivariate analysis was performed, including variables with *p*-value below 0.2 on univariate analysis, *i*.*e*. the presence of jaundice, a total bilirubin level equal to or above 40 μmol/L, a leukocyte count greater than 7.9x10^9^/L and a reticulocyte count above182x10^9^/L. The parameters that were associated with VTE were a leukocyte count above 7.9x10^9^/L (odds ratio (OR) = 15.7; *p* = 0.02) and a total bilirubin level of 40 μmol/L or above (OR = 7.4; *p* = 0.02).

**Fig 2:**
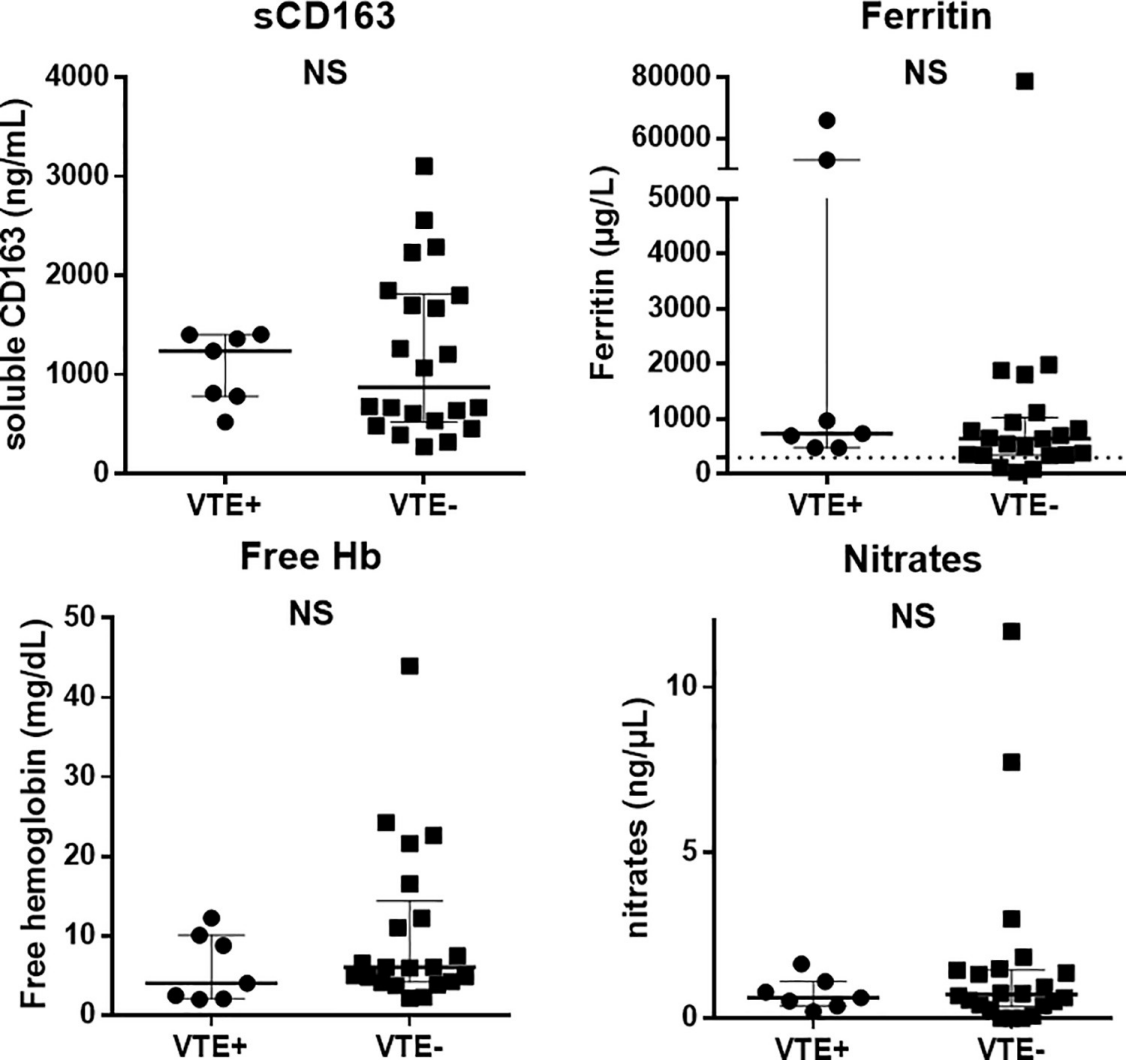
Comparison of serum levels of soluble CD163, ferritin, free hemoglobin and nitrates between patients with venous thromboembolism (VTE+) and those without (VTE-). Results are given by medians [interquartile range (IQR)]. NS: non-significant.

In order to determine predictive factor of thrombosis, the levels of soluble CD163, free hemoglobin and NO products were retrospectively measured on serum frozen at wAIHA diagnosis in 7 patients with VTE and 22 patients without VTE. These parameters were specifically studied due to their involvement in hemolysis. Indeed, free hemoglobin released during hemolysis binds to haptoglobin that will subsequently bind to its receptor (CD163) on activated macrophages [23]. As shown in hereditary or acquired hemolytic anemias [10,24,25], once this mechanism is saturated due to high hemolysis rate, the increase in free hemoglobin in the plasma scavenges NO, thus resulting in a prothrombotic state, due to vasoconstriction, activation of endothelial cells and platelet aggregation. However, none of these parameters were significantly different between the two groups (Fig 2).

## Discussion

In our study, 23% of patients with primary or secondary wAIHA experienced at least one episode of VTE during the follow-up period. This result is in keeping with the data from the literature, with thrombosis reported to occur in 11 to 27% [8,13,14,26–29]. It is worth noticing that the primary or secondary nature of the AIHA did not influence the risk of thrombosis, which corroborates a previous report in a cohort of 60 patients [28].

During AIHA, the thrombotic risk is reported to not correlate with traditional risk factors [14], which was confirmed in our study, and stresses the fact that the risk of VTE in young patients with wAIHA should not be underestimated. Moreover, VTE was consistent with PE in more than half of the cases, as previously reported [14], and it is possible that in daily clinical practice, symptoms of PE could be wrongly attributed to anemia, thus leading to an underestimation of VTE or to a delay in its diagnosis. It also suggests that AIHA carries an inherent risk of thrombosis. Hemolysis by itself is likely to be an independent risk factor for thrombosis, as hemolysis was present at the time of VTE in 9 out of 10 patients, which corroborates previous reports [6,14,28,30]. Moreover, hemolytic parameters such as total bilirubin level and reticulocyte count at the diagnosis of wAIHA were higher in patients with VTE, which is in line with the higher level of LDH observed in the largest series of primary AIHA [13]. Contrary to others [13,14], we did not find that patients with VTE had a more profound anemia at diagnosis or during VTE compared to those without. Overall, in such a context of wAIHA, nor the presence of icterus, the level of bilirubin, the level of LDH, reticulocyte count, nor the level of hemoglobin at AIHA diagnosis or during follow-up appeared as clinically relevant parameters to screen for patients with the higher risk of thrombosis. As previously notified by others [13,14], there is a need to determine predictive factors of thrombosis during AIHA, as classical predictive factors are not sufficient. For this reason, we investigated the levels of sCD163, free hemoglobin and NO metabolites, but no difference was observed between patients with or without VTE. More recently, it has been shown that heme participates to granulocyte migration and to the release of neutrophil extracellular traps (NET), that could contribute to VTE by recruiting platelets and increasing their activation and thrombin generation [31]. These phenomena could account for the fact that in our study, a high leucocyte count was associated with VTE.

The favoring role of antiphospholipid antibodies has been reported in several studies [6,27], but was not confirmed in others [13,14,28], including our study. Splenectomy has also been reported as an independent risk factor for thrombosis during AIHA [8,13]. In our study, splenectomy was performed in 9 patients among which 2 had portal system VTE postoperatively.

VTE occurred within the 8 weeks following the initial diagnosis in 9 out of 10 patients, which is in line with data from the literature that reports a maximal risk of thrombosis within the 3 months following the diagnosis [4]. For 8 patients, VTE occurred within the 48h following hospital admission, in the absence of prophylactic anticoagulation. This corroborates the results of Lecouffe-Desprets *et al*. who observed VTE in 6/8 outpatients with low Padua score [14]. The importance of thromboprophylaxis during AIHA has already been pointed out in a study in which 5 episodes of thrombosis were observed during 15 hemolytic flares without prophylactic anticoagulation, compared with one out of 21 flares occurring in patients with preventive anticoagulation [30]. Moreover, due to the occurrence of a severe VTE after hospital discharge, the authors proposed not to discontinuing preventive anticoagulation until hemolysis is stopped. In our study, VTE occurred after hospital discharge in 4 patients with persistent hemolysis and was probably favored by other factors such as bedrest, IVIg infusion or nephrotic syndrome. Although the reluctance to prescribe an anticoagulation therapy in patients with anemia is understandable, once the hemolytic etiology is confirmed, prophylactic anticoagulation should not be delayed, even in the absence of common VTE risk factors. Moreover, the maintenance of thromboprophylaxis after hospital discharge should be discussed on an individual basis, depending on the persistence of hemolysis and risk factors of thrombosis. Aspirin has been proposed by authors to prevent VTE [10,32], which was not supported by our results, as the use of antiplatelet therapy was similar between patients with or without VTE.

Importantly, our results confirmed that the occurrence of VTE does not increase mortality rate during wAIHA [13], contrary to what was initially reported [8], probably due to improvement in the therapeutic management of VTE.

Our study has some limitations. Its retrospective design could have been a source of bias, especially for the determination of the Wells and Padua prediction scores. A predisposing factor such as bedrest for more than 72h could have been missed in the medical record, leading to an underestimation of the VTE risk for patients with active hemolysis. The sample size was rather small, which may have affected the power of the study. Nonetheless, except for the Italian series that included 308 primary AIHA [13], similar studies in the literature rarely included more than 50 patients, as wAIHA is a rare disease.

## Conclusions

In this retrospective study, VTE occurred in 1 out of 5 patients, at a similar frequency in primary and secondary wAIHA. No predictive factor of VTE could be identified, apart from a high bilirubin level. Nonetheless, these results are in agreement with other studies, and suggest that the risk of thrombosis increases with the intensity of hemolysis, supported by the fact that VTE mostly occurred in the weeks following the diagnosis of AIHA. For the first time, we also observed a higher leucocyte count in patients with VTE, which could argue for the participation of leukocytes in VTE pathogenesis or depict a higher bone marrow regeneration.

Due to the occurrence of VTE early in the course of wAIHA and the absence of specific criteria to identify the patients with the higher risk, we do think that a systematic clinical screening for VTE must be performed at AIHA diagnosis. We also recommend to promptly initiate a prophylactic anticoagulation once the diagnosis of wAIHA is confirmed, regardless other thrombotic risk factors. Moreover, the maintenance of thromboprophylaxis after hospital discharge may be discussed until hemolysis is controlled but also depending on other risk factors of thrombosis.

## Supporting information

S1 Table Patients’ characteristics. (XLSX)

## References

[1] Zoller B, Li X, Sundquist J, Sundquist K (2012) Risk of pulmonary embolism in patients with autoimmune disorders: a nationwide follow-up study from Sweden. Lancet 379: 244–249. 10.1016/S0140-6736(11)61306-8 22119579

[2] Ramagopalan SV, Wotton CJ, Handel AE, Yeates D, Goldacre MJ (2011) Risk of venous thromboembolism in people admitted to hospital with selected immune-mediated diseases: record-linkage study. BMC Med 9: 1 10.1186/1741-7015-9-1 21219637 PMC3025873

[3] Johannesdottir SA, Schmidt M, Horvath-Puho E, Sorensen HT (2012) Autoimmune skin and connective tissue diseases and risk of venous thromboembolism: a population-based case-control study. J Thromb Haemost 10: 815–821. 10.1111/j.1538-7836.2012.04666.x 22353382

[4] Yusuf HR, Hooper WC, Grosse SD, Parker CS, Boulet SL, Ortel TL (2015) Risk of venous thromboembolism occurrence among adults with selected autoimmune diseases: A study among a U.S. cohort of commercial insurance enrollees. Thromb Res 135: 50–57. 10.1016/j.thromres.2014.10.012 25456001 PMC4480419

[5] Zoller B, Li X, Sundquist J, Sundquist K (2012) Autoimmune diseases and venous thromboembolism: a review of the literature. Am J Cardiovasc Dis 2: 171–183. 22937487 PMC3427982

[6] Pullarkat V, Ngo M, Iqbal S, Espina B, Liebman HA (2002) Detection of lupus anticoagulant identifies patients with autoimmune haemolytic anaemia at increased risk for venous thromboembolism. Br J Haematol 118: 1166–1169. 12199802 10.1046/j.1365-2141.2002.03729.x

[7] van Zaane B, Nur E, Squizzato A, Gerdes VE, Buller HR, Dekkers OM, et al (2010) Systematic review on the effect of glucocorticoid use on procoagulant, anti-coagulant and fibrinolytic factors. J Thromb Haemost 8: 2483–2493. 10.1111/j.1538-7836.2010.04034.x 20735729

[8] Allgood JW, Chaplin H, Jr. (1967) Idiopathic acquired autoimmune hemolytic anemia. A review of forty-seven cases treated from 1955 through 1965. Am J Med 43: 254–273. 6034957 10.1016/0002-9343(67)90168-4

[9] Ataga KI (2009) Hypercoagulability and thrombotic complications in hemolytic anemias. Haematologica 94: 1481–1484. 10.3324/haematol.2009.013672 19880774 PMC2770956

[10] L'Acqua C, Hod E (2015) New perspectives on the thrombotic complications of haemolysis. Br J Haematol 168: 175–185. 10.1111/bjh.13183 25307023

[11] Cappellini MD (2007) Coagulation in the pathophysiology of hemolytic anemias. Hematology Am Soc Hematol Educ Program: 74–78. 10.1182/asheducation-2007.1.74 18024612

[12] Ungprasert P, Tanratana P, Srivali N (2015) Autoimmune hemolytic anemia and venous thromboembolism: A systematic review and meta-analysis. Thromb Res 136: 1013–1017. 10.1016/j.thromres.2015.09.004 26359320

[13] Barcellini W, Fattizzo B, Zaninoni A, Radice T, Nichele I, Di Bona E, et al (2014) Clinical heterogeneity and predictors of outcome in primary autoimmune hemolytic anemia: a GIMEMA study of 308 patients. Blood 124: 2930–2936. 10.1182/blood-2014-06-583021 25232059

[14] Lecouffe-Desprets M, Neel A, Graveleau J, Leux C, Perrin F, Visomblain B, et al (2015) Venous thromboembolism related to warm autoimmune hemolytic anemia: a case-control study. Autoimmun Rev 14: 1023–1028. 10.1016/j.autrev.2015.07.001 26162301

[15] Hochberg MC (1997) Updating the American College of Rheumatology revised criteria for the classification of systemic lupus erythematosus. Arthritis Rheum 40: 1725. 10.1002/art.1780400928 9324032

[16] Lundberg IE, Tjarnlund A, Bottai M, Werth VP, Pilkington C, de Visser M, et al (2017) 2017 European League Against Rheumatism/American College of Rheumatology Classification Criteria for Adult and Juvenile Idiopathic Inflammatory Myopathies and Their Major Subgroups. Arthritis Rheumatol 69: 2271–2282. 10.1002/art.40320 29106061 PMC5846474

[17] Swerdlow SH, Campo E, Pileri SA, Harris NL, Stein H, Siebert R, et al (2016) The 2016 revision of the World Health Organization classification of lymphoid neoplasms. Blood 127: 2375–2390. 10.1182/blood-2016-01-643569 26980727 PMC4874220

[18] Arber DA, Orazi A, Hasserjian R, Thiele J, Borowitz MJ, Le Beau MM, et al (2016) The 2016 revision to the World Health Organization classification of myeloid neoplasms and acute leukemia. Blood 127: 2391–2405. 10.1182/blood-2016-03-643544 27069254

[19] Rajkumar SV, Dimopoulos MA, Palumbo A, Blade J, Merlini G, Mateos MV, et al (2014) International Myeloma Working Group updated criteria for the diagnosis of multiple myeloma. Lancet Oncol 15: e538–548. 10.1016/S1470-2045(14)70442-5 25439696

[20] Barbar S, Noventa F, Rossetto V, Ferrari A, Brandolin B, Perlati M, et al (2010) A risk assessment model for the identification of hospitalized medical patients at risk for venous thromboembolism: the Padua Prediction Score. J Thromb Haemost 8: 2450–2457. 10.1111/j.1538-7836.2010.04044.x 20738765

[21] Vardi M, Ghanem-Zoubi NO, Zidan R, Yurin V, Bitterman H (2013) Venous thromboembolism and the utility of the Padua Prediction Score in patients with sepsis admitted to internal medicine departments. J Thromb Haemost 11: 467–473. 10.1111/jth.12108 23279085

[22] Crosby WH, Furth FW (1956) A modification of the benzidine method for measurement of hemoglobin in plasma and urine. Blood 11: 380–383. 13304127

[23] Moestrup SK, Moller HJ (2004) CD163: a regulated hemoglobin scavenger receptor with a role in the anti-inflammatory response. Ann Med 36: 347–354. 15478309 10.1080/07853890410033171

[24] Peacock-Young B, Macrae FL, Newton DJ, Hill A, Ariens RAS (2018) The prothrombotic state in paroxysmal nocturnal hemoglobinuria: a multifaceted source. Haematologica 103: 9–17. 10.3324/haematol.2017.177618 29246924

[25] Hill A, Kelly RJ, Hillmen P (2013) Thrombosis in paroxysmal nocturnal hemoglobinuria. Blood 121: 4985–4996; quiz 5105. 10.1182/blood-2012-09-311381 23610373

[26] Bongarzoni V, Annino L, Roveda A, Amendolea MA, Tirindelli MC, Avvisati G (2005) Risk of thromboembolism in patients with idiopathic autoimmune hemolytic disease and antiphospholipid antibodies: results from a prospective, case-control study. Haematologica 90: 711–713. 15929203

[27] Kokori SI, Ioannidis JP, Voulgarelis M, Tzioufas AG, Moutsopoulos HM (2000) Autoimmune hemolytic anemia in patients with systemic lupus erythematosus. Am J Med 108: 198–204. 10723973 10.1016/s0002-9343(99)00413-1

[28] Roumier M, Loustau V, Guillaud C, Languille L, Mahevas M, Khellaf M, et al (2014) Characteristics and outcome of warm autoimmune hemolytic anemia in adults: New insights based on a single-center experience with 60 patients. Am J Hematol 89: E150–155. 10.1002/ajh.23767 24847759

[29] Baek SW, Lee MW, Ryu HW, Lee KS, Song IC, Lee HJ, et al (2011) Clinical features and outcomes of autoimmune hemolytic anemia: a retrospective analysis of 32 cases. Korean J Hematol 46: 111–117. 10.5045/kjh.2011.46.2.111 21747883 PMC3128891

[30] Hendrick AM (2003) Auto-immune haemolytic anaemia—a high-risk disorder for thromboembolism? Hematology 8: 53–56. 10.1080/1024533021000059474 12623428

[31] Chen G, Zhang D, Fuchs TA, Manwani D, Wagner DD, Frenette PS (2014) Heme-induced neutrophil extracellular traps contribute to the pathogenesis of sickle cell disease. Blood 123: 3818–3827. 10.1182/blood-2013-10-529982 24620350 PMC4055928

[32] Weinkle TK, Center SA, Randolph JF, Warner KL, Barr SC, Erb HN (2005) Evaluation of prognostic factors, survival rates, and treatment protocols for immune-mediated hemolytic anemia in dogs: 151 cases (1993–2002). J Am Vet Med Assoc 226: 1869–1880. 15934255 10.2460/javma.2005.226.1869

